# Electrophysiological Evidence for Functional (Psychogenic) Essential Palatal Tremor

**DOI:** 10.5334/tohm.70

**Published:** 2020-06-17

**Authors:** Felipe Vial, Emmanuel Akano, Sanaz Attaripour, Patrick McGurrin, Mark Hallett

**Affiliations:** 1 National Institutes of Health, NINDS, Human Motor Control Section, Bethesda, Maryland, US; 2 Universidad del Desarrollo, Facultad de Medicina Clínica Alemana, Av Vitacura, Vitacura, Región Metropolitana, CL; 3 National Institutes of Health, NINDS, Molecular Neuropharmacology Unit, Bethesda, MD, US; 4 Department of Neurology, University of California, Irvine, California, US

**Keywords:** tremor, functional, psychogenic, palatal myoclonus, Bereitschaftspotential

## Abstract

**Background:**

There is little published work describing the electrophysiological characteristics of essential palatal tremor, a condition now believed by many to be a functional (psychogenic) movement disorder.

**Case Report:**

Here we combine electroencephalography and electromyography with time-locked video recordings to document two cases of essential palatal tremor in which a definitive diagnosis is achieved using these electrophysiological tools.

**Discussion:**

We believe that sharing how these objective tools can be used to diagnose a functional movement disorder, as well as providing more published evidence to support the functional origin of essential palatal myoclonus, will help to diagnose this condition in the future.

## Introduction

Palatal tremor (PT) or palatal myoclonus is an uncommon hyperkinetic movement disorder characterized by a rhythmic 0.5–3 Hz palatal movement. It is classified into two groups; essential and symptomatic PT. Essential PT is produced by contraction of the tensor veli palatini muscle and is usually associated with an ear clicking sound but no other neurological signs. Symptomatic PT is produced by contraction of the levator veli palatini muscle and is associated with ataxia and pendular nystagmus [3].

It is now thought by many that most essential PT patients are functional (psychogenic). This has been supported by the fact that the movement in these cases is incongruous, variable, entrainable, and distractible on electromyographic (EMG) recordings [7]. Here we present two cases of essential PT in which simultaneous electroencephalography (EEG) and EMG activity were recorded to explore how electrophysiology can be used in different ways to understand the functional etiology of this condition.

### Clinical case 1

A 48-year-old male had a 6-year history of involuntary palatal movements, with the onset occurring after a plane trip. The movements coincide with a clicking sound, are constant throughout the day, and only disappear when he is speaking or eating. He reports being able to withhold the palatal movement, but only for about 10 seconds. He did not report any urge before or relief after the movement, and there was no history of childhood tics. He has tried clonazepam, diazepam, lamotrigine, and levetiracetam without benefit. He has also tried botulinum toxin injections with no benefit.

His examination was remarkable for a 1–2 Hz semi-rhythmic, symmetric, slow soft-palatal elevation, together with a click sound that disappeared when asked to say a prolonged “a.” There was no clear distractibility on the examination. The rest of the exam, in particular, the eye movements and cerebellar function, was normal. A brain MRI with thin cuts at the level of the inferior olivary nucleus and cerebellar regions was normal. The history and examination were compatible with the diagnosis of essential PT.

About 3 years after the beginning of the symptoms, he took Adderall with the purpose of staying awake and alert for more extended periods of time. However, he realized that there were no more palatal movements. He self-reported that this effect lasts for about 6 hours. Since that time, he has been taking Adderall XR (60 mg once a day).

### Clinical case 2

46-year-old male had a 6-year history of involuntary palatal movements, with the onset occurring about two months after a protracted dental procedure for gum resection (gingival graft). Initially, he had a sense of fullness in both ears – worse on the right side – which persisted for a few months. As this sense of ear fullness subsided, he developed involuntary movements of the palate, and concurrent ear clicks that he feels are constant throughout the day, even while speaking or swallowing. He can transiently suppress the movement for about 20–30 seconds. He did not report any urge before or relief after the movement. There was no history of childhood tics.

Examination revealed a semi-rhythmic 3 Hz palatal movement originating from the roof of the soft palate, together with a click sound that disappeared when asked to say a prolonged “a.” Also, we did not observe fluctuations in the voice while the patient was speaking, arguing against the history of constant palatal movements while the patient was speaking. The palatal tremors entrained to different frequencies of hand taps. The rest of his neurological examination, including cerebellar and eye movement examinations, were unremarkable. There were no cerebellar, cognitive, or other neurological symptoms.

Audiometry and indirect laryngoscopy at another institution did not reveal any abnormalities. MRI of the brain revealed no brainstem or cerebellar lesions, nor olivary pseudohypertrophy. A diagnosis of essential PT was made.

He has not taken any medications for his symptoms but does note that the movements stop for a few seconds to minutes while meditating. Intermittent facial grimacing and side-to-side jaw movements were noted during meditation.

## Electrophysiological study

For both cases, an electrophysiological study, including EMG and EEG, was conducted to further characterize the movement. Sticky surface EMG electrodes were stuck to the soft and hard palate. In addition, EEG activity was measured over Cz with reference to left mastoid and ground on FP1. Surface EMG was also placed on the left abductor pollicis brevis (APB) muscle to record voluntary movements to assess distractibility and entrainment. Simultaneous EMG and EEG recordings were done during rest (with mouth open and closed) and distraction maneuvers, namely tapping with the left hand.

**Figure 1:**
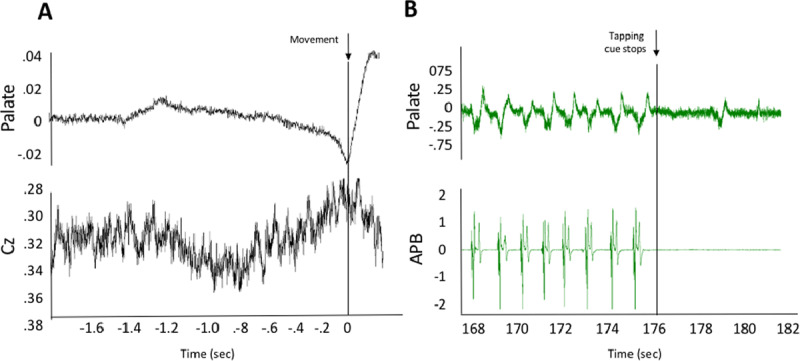
**Electrophysiology study – case 1. (A)** Average of 74 traces from simultaneous electromyography (EMG; top) and electroencephalographic (EEG; bottom) recordings. Markers were manually placed at the onset of each movement of the palate, and all movements were subsequently averaged. A Bereitschaftspotential (BP) is visible starting roughly 800 ms prior to movement onset. **(B)** An example of simultaneous EMG recordings of the palate (top) and APB (bottom) muscles during left-hand tapping at 1 Hz. The palatal movement is entrained at 1 Hz, and at the offset of tapping there is a clear change in the frequency of the palatal tremor.

In case 1, we were able to identify a clear Bereitschaftspotential (BP) by back-averaging to the palatal movement (Figure 1a). The back-averaging technique is described elsewhere [8]. Distractibility and entrainment were also observed when tapping at 1 Hz. (Figure 1b). The movement entrained to the tapping frequency and also paused at the end of the tapping task.

**Figure 2:**
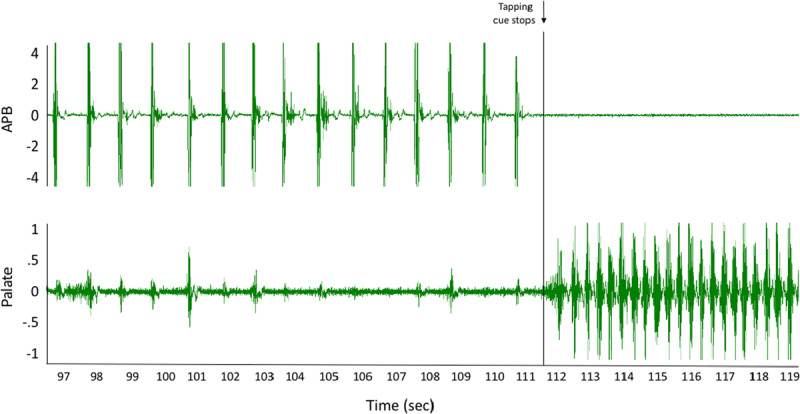
**Electrophysiology study – case 2.** An example of simultaneous electromyography (EMG) recordings of the APB (top) and palate (bottom) muscles during left-hand tapping at 1 Hz. The palatal movement is entrained at 1 Hz, and at the offset of tapping there is a clear change in the frequency of the tremor back to 3 Hz.

In case 2, we also saw entrainment when tapping at 1 Hz. (Figure 2; Video 1 with synchronous EMG in supplemental material). At the offset of tapping, there was a clear change in the frequency of the tremor back to 3 Hz. In this case we do not did not find a BP after backaveraging.

**Video 1:** **Time-locked video and Electromyographic recordings – case 2.** Electromyography (EMG) and time-locked video from case 2 demonstrating entrainment of the palate to tapping. The EMG data correspond to that shown in Figure 2. Vertical lines in the video indicate when the recording begins (“video begins”), as well as when tapping with APB stopped (“Tapping stops”). EMG alignment to the video can be appreciated by observing the right side of the EMG traces as they enter the viewing screen.

## Discussion

Together, these two cases highlight how electrophysiology can be used in different ways to document a functional syndrome. Case 1 showed a BP, a slowly rising negative potential originating over the premotor and supplementary motor areas that has been shown to precede both voluntary and functional movements [4]. In this situation, the presence of BP is a positive indication of a functional syndrome. We did not find a BP in case 2, and this might well be due to the high frequency of the movements, which make BP recording and interpretation difficult given the 1–2 sec duration of a BP. This failure certainly shows that although very useful when present, BPs can be technically difficult to record. This is a limitation of the technique. In both cases, however, we were able to demonstrate the functional origin of the movement by showing the distractibility and entrainment with other electrophysiological tools. To our knowledge, there is only one previous report of an electrophysiological demonstration of BPs in essential palatal tremor [9]. There is also a report of a case of essential palatal tremor in which the electrophysiology gives support to an organic cause [6]. In this patient the tremor frequency remained constant during entrainment testing.

Finding one or more positive features, and avoiding reliance solely on exclusion of other conditions, is critical for the diagnosis of functional movement disorders [2]. As shown in these cases, the electrophysiology can help give positive, objective measures to the diagnosis. Having a firm diagnosis is critical to avoid costly, unnecessary studies and futile pharmacological or even surgical interventions [5]. In our experience, these data also provide a visual tool that is helpful for explaining the diagnosis to the patients and engaging them in their treatment.

There is significant literature describing the importance of attention in the development and maintenance of somatic symptoms [1]. The fact that these patients’ symptoms responded significantly to Adderall and meditation, respectively, supports this role of attention in the pathogenesis of functional movement disorder (FMD). Furthermore, it may support how shifting between different attention modules can be a potential source for treatment. This hypothesis needs to be further investigated in pre-clinical and clinical studies.

## Appendix

**Table:**

Name	Location	Role	Contribution

Felipe Vial	National Institutes of Health, NINDS	Author	Acquisition and interpretation of the data, writing the report
Emmanuel Akano	National Institutes of Health, NINDS	Author	Acquisition and interpretation of the data, writing the report
Patrick McGurrin	National Institutes of Health, NINDS	Author	Acquisition and interpretation of the data
Sanaz Attaripour	National Institutes of Health, NINDS	Author	Acquisition and interpretation of the data
Mark Hallett	National Institutes of Health, NINDS	Author	Writing the report
